# An Unusual Case of Genital Myiasis in Nicaragua: A Multidisciplinary Approach in a Patient With Stage IV Pelvic Organ Prolapse

**DOI:** 10.7759/cureus.83411

**Published:** 2025-05-03

**Authors:** María Esther Suárez Garcia, Andres Rivera, Jasser Logo Cabrera, Ramon Roque, Jorge Cristhian Ocon Espinoza, Christopher Kaleb Romero Ríos

**Affiliations:** 1 Obstetrics and Gynecology, Hospital Militar Escuela "Dr. Alejandro Dávila Bolaños", Managua, NIC; 2 School of Medicine, Hospital Militar Escuela "Dr. Alejandro Dávila Bolaños", Managua, NIC

**Keywords:** multidisciplinary management, myiasis infestation, pelvic organ prolapse, postmenopausal female, surgical debridement

## Abstract

Myiasis, a parasitic infestation caused by fly larvae in necrotic tissue, is rare in urban settings but can complicate conditions like advanced pelvic organ prolapse (POP). This report highlights the diagnostic and therapeutic challenges of myiasis in a patient with grade IV POP. An 85-year-old postmenopausal woman presented with a four-year history of vaginal mass protrusion, recent bleeding, foul discharge, and necrotic tissue. Examination revealed grade IV POP with left-sided necrotic tissue (4 × 4 cm) infested with larvae. Laboratory findings showed leukocytosis (17.57 × 10³/µL), elevated CRP (28.03 mg/dL), and a hemoglobin level of 14 g/dL. Management included surgical debridement, saline/chlorhexidine lavage, ivermectin, and broad-spectrum antibiotics (ceftriaxone/clindamycin), later adjusted to cefepime after cultures grew *Escherichia coli* and *Sphingomonas paucimobilis*. Imaging revealed bladder stones treated with pneumatic cystolithotripsy. Definitive surgery (vaginal hysterectomy, colpectomy, and perineoplasty) resolved the prolapse. This case emphasizes the importance of multidisciplinary care, early debridement, tailored antibiotics, and definitive surgery in managing complex myiasis with POP. It underscores the need for hygiene education and regular follow-up in high-risk patients.

## Introduction

Myiasis, the parasitic infestation of human tissues by fly larvae, primarily occurs in necrotic or decomposing tissue [1]. Although more prevalent in tropical regions, urban cases are increasingly reported, especially among populations with open wounds, suboptimal hygiene practices, or immunosuppressive conditions [2]. While cutaneous manifestations dominate clinical reports, genital and pelvic myiasis remains exceptionally rare, with only sporadic cases associated with advanced pelvic organ prolapse (POP) [3]. Grade IV uterine prolapse, defined by complete extrusion of the uterus beyond the vaginal introitus, generates a microenvironment of chronic tissue ischemia, mucosal ulceration, and bacterial colonization, creating a nidus for secondary infections [4]. Reports of myiasis complicating POP are strikingly limited, and current management protocols lack standardization, often requiring multimodal strategies such as mechanical larval extraction, antimicrobial therapy, and definitive surgical correction of anatomical defects [5]. The confluence of prolonged tissue exposure, inadequate wound care, and environmental factors (e.g., warm climates or poor sanitation) heightens susceptibility to infestation; nonetheless, fewer than 10 cases of POP-associated myiasis have been documented worldwide, reflecting its status as a medical rarity [3,6].

This case contributes to the sparse literature by detailing a multidisciplinary approach to managing myiasis in grade IV POP, a scenario with unique anatomical and logistical challenges. It highlights the critical need for early recognition, aggressive debridement, and tailored surgical planning to mitigate life-threatening complications such as sepsis or systemic larval migration. By synthesizing existing evidence with novel clinical insights, this report aims to inform future guidelines for high-risk populations and reinforce the importance of integrating parasitological management into gynecologic care.

## Case presentation

An 85-year-old woman with infected grade IV POP type C presented to the ED with a five-day history of scant vaginal bleeding, a protruding genital mass (present for four years), throbbing pelvic pain, severe perineal itching, and foul-smelling discharge. She lived in an urban area, denied trauma or insect bites, and had no significant medical history. Her gynecological background included menarche at age 14, multiple pregnancies, menopause at 52, and no prior cervical cancer screenings.

**Figure 1:**
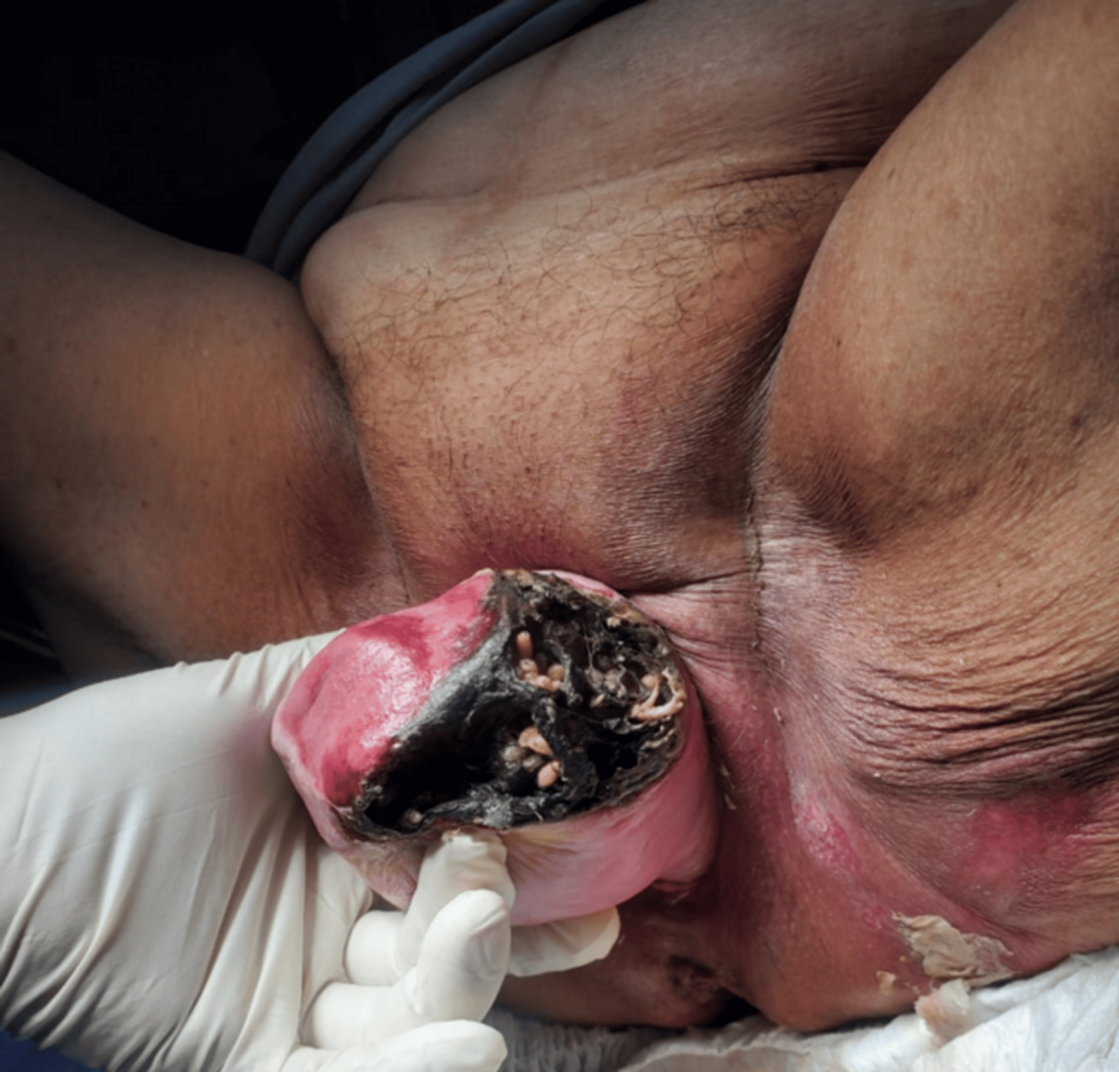
Myiasis (larval infestation) in necrotic prolapsed tissue

On examination, she was hemodynamically stable and alert. Gynecological evaluation revealed a grade IV POP with complete cervical protrusion (+6 cm beyond the vaginal introitus) and a 4 × 4 cm area of necrotic, malodorous tissue on the left side (Figure 1). Laboratory findings included leukocytosis (17.57 × 10³/µL, 82.1% neutrophils), elevated CRP (28.03 mg/dL), and normal renal, coagulation, and electrolyte panels.

**Figure 2:**
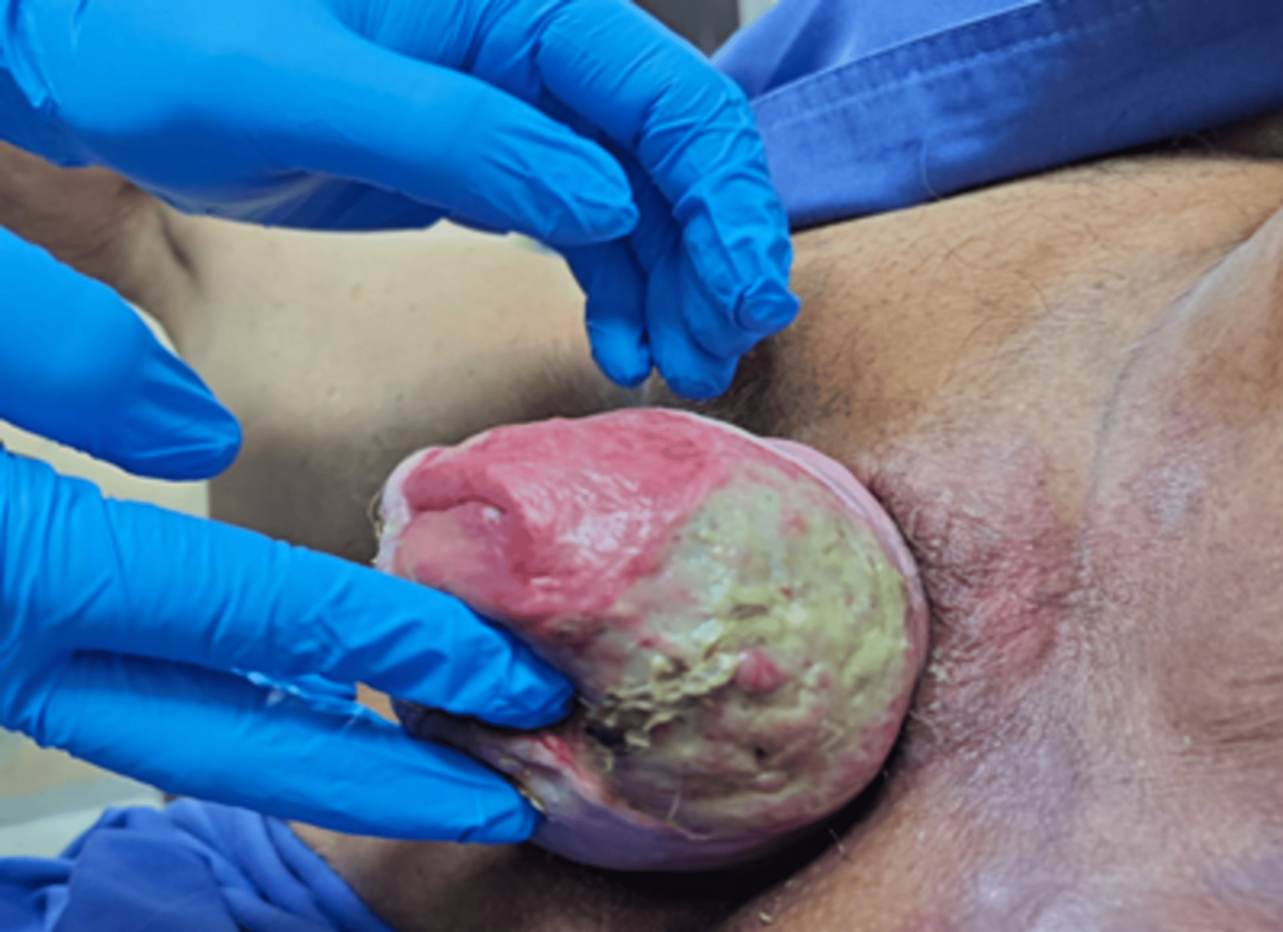
Grade IV POP with fibrin deposits post-debridement POP, pelvic organ prolapse

She underwent urgent surgical debridement. Intraoperatively, grade IV POP with myiasis (fly larvae infestation) was confirmed, alongside extensive left-sided necrotic tissue. Secretion cultures and a Pap smear were collected. Necrotic tissue was excised, followed by rigorous irrigation with saline and 4% chlorhexidine to remove larvae. Minimal bleeding was noted. Postoperatively, she received broad-spectrum antibiotics (ceftriaxone and clindamycin) and oral ivermectin, managed by a multidisciplinary team. A second debridement revealed persistent necrotic tissue (7 × 4 cm), which was irrigated again (Figure 2).

**Figure 3:**
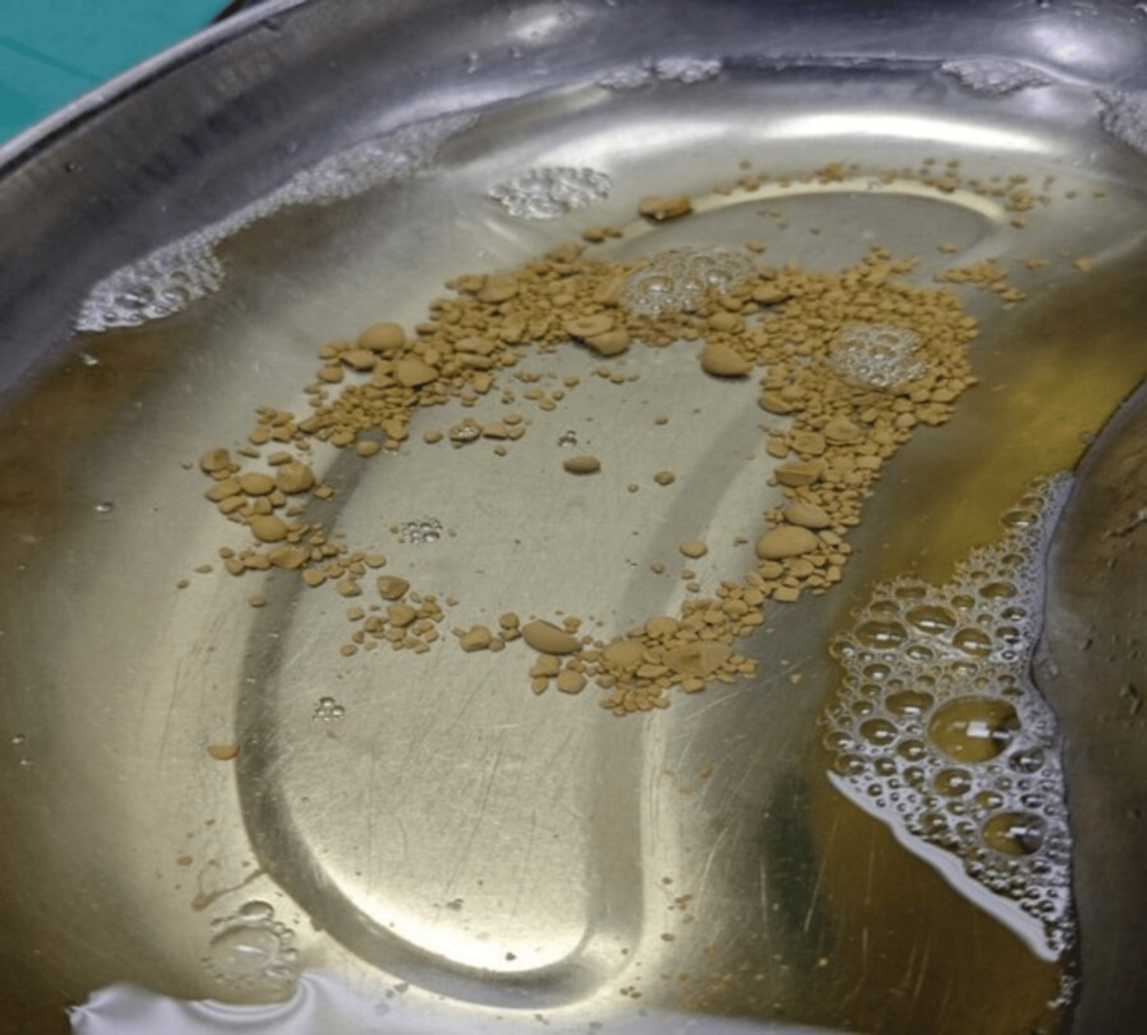
Bladder stones removed via lithotripsy

Microbial cultures identified *Escherichia coli *and *Sphingomonas paucimobilis*, prompting a switch to cefepime as antibiotic therapy. Initial management did not include hysterectomy due to suspicion of vesical myiasis suggested by pelvic MRI, which revealed bladder prolapse and multiple rounded calcifications (2-18 mm). Subsequent cystoscopy confirmed the presence of bladder stones, with the largest calculus measuring 15 mm in diameter, treated via pneumatic lithotripsy and Foley catheter placement (Figure 3). Post-procedure, a 7 × 5 cm fibrinous tissue in the prolapsed area was irrigated.

**Figure 4:**
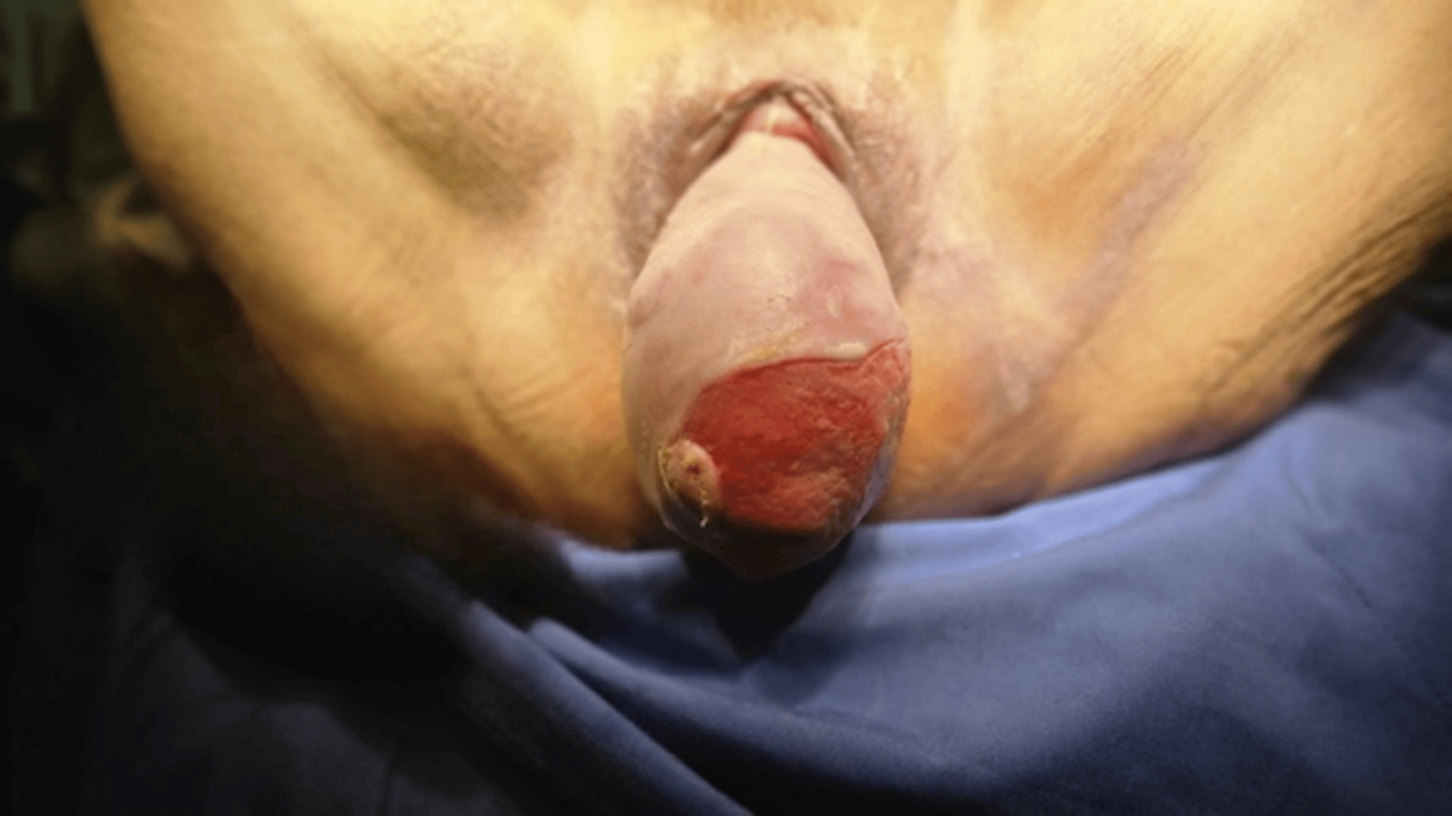
Severe prolapse prior to definitive surgery

Twice-daily wound care with chlorhexidine, saline, and ketanserin gel promoted healing, resolving infection and larval presence but leaving residual fibrin. After two surgical lavages, definitive surgery - vaginal hysterectomy, colpectomy, and perineoplasty - was performed. Intraoperatively, friable scar tissue and fibrosis (7 × 4 cm) were noted (Figure 4).

**Figure 5:**
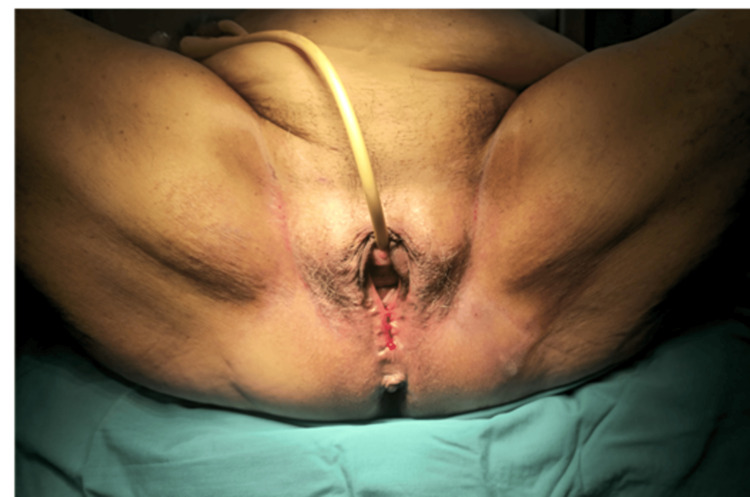
Postoperative anatomical restoration

The patient recovered well post-surgery (Figure 5). Her postoperative course was marked by steady improvement, including stable hemodynamics and optimal wound healing. Based on these positive outcomes, she was cleared for discharge 72 hours after the procedure. A follow-up evaluation was arranged within 48 hours via the outpatient clinic to monitor recovery, evaluate anatomical repair, and address any emerging concerns, reinforcing a patient-centered transition from hospital to home care.

## Discussion

Severe uterine prolapse in postmenopausal women leads to ischemia, ulceration, and secondary infection, conditions that may attract insect vectors of myiasis [2]. Myiasis, a parasitic infestation caused by fly larvae in living tissues, typically occurs in settings of poor hygiene, open wounds, or tissue necrosis. Its manifestation in the genital tract is rare and poses diagnostic and therapeutic challenges. In this case, a patient with grade IV POP developed superimposed myiasis, complicating her clinical course and necessitating multidisciplinary intervention [7].

Therapeutic management of myiasis involves larval removal, surgical debridement of necrotic tissue, and empiric antibiotics to prevent secondary bacterial infections [8]. Here, initial treatment included broad-spectrum antibiotics, later adjusted after cultures identified *E. coli* and *S. paucimobilis *- opportunistic pathogens capable of causing severe infections in immunocompromised patients or those with devitalized tissue [9].

A notable finding in this case was bladder lithiasis, detected by MRI, which raised suspicion of vesical myiasis. Although rare, larval infestation of the urinary tract has been reported in patients with immunosuppression or poor hygiene. Surgical resolution through cystolithotripsy ruled out bladder larvae and improved the patient’s prognosis [10].

Definitive treatment included vaginal hysterectomy, colpectomy, and perineoplasty - well-established techniques for advanced uterine prolapse. Vaginal hysterectomy is a safe and effective procedure for prolapse correction, minimizing surgical trauma and accelerating recovery [11]. The Rouhier colpocleisis technique, combining vaginal hysterectomy with vaginal closure, is used to manage POP associated with uterine pathology [12].

In this 85-year-old patient with grade IV POP and myiasis, colpohysterectomy effectively resolved the prolapse and associated complications. The procedure enabled necrotic tissue removal and pelvic anatomy restoration, enhancing the patient’s quality of life [13]. It is important to highlight that the choice of surgical technique should be individualized, taking into account factors such as the patient’s age, comorbidities, desire to preserve sexual function, and the presence of other medical conditions. In elderly patients or those at high surgical risk, colpocleisis, which involves the partial or total closure of the vaginal cavity, may be an appropriate alternative [14,15].

Medical literature on myiasis complicating uterine prolapse remains scarce. This case underscores the need to document and study such rare presentations to refine clinical and preventive strategies. Hygiene education and regular medical follow-up in women with advanced POP are critical to preventing this condition [12].

## Conclusions

Myiasis in the context of advanced POP is a rare yet potentially severe complication. In this case, prompt identification and multidisciplinary management ensured a favorable outcome, underscoring the critical role of timely diagnosis and definitive surgical intervention. The presence of secondary bacterial infections and suspected vesical myiasis (bladder involvement) highlights the complexity of such cases and the necessity for rigorous clinical vigilance.

Given the limited literature on this condition, this case enriches clinical understanding and emphasizes the importance of preventive measures, including proper hygiene and regular medical follow-up in patients with severe uterine prolapse. Documenting such cases is vital to refining future clinical management and enhancing care protocols, ensuring optimized outcomes for similar presentations.
